# Implementation of new standard operating procedures for geriatric trauma patients with multiple injuries: a single level I trauma centre study

**DOI:** 10.1186/s12877-019-1380-z

**Published:** 2019-12-19

**Authors:** Lorenz Peterer, Christian Ossendorf, Kai Oliver Jensen, Georg Osterhoff, Ladislav Mica, Burkhardt Seifert, Clément M. L. Werner, Hans-Peter Simmen, Hans-Christoph Pape, Kai Sprengel

**Affiliations:** 1 Department of Trauma, University Hospital Zurich, Raemistrasse 100, 8091 Zurich, Switzerland; 2 Department of Biostatistics at Epidemiology, Biostatistics and Prevention Institute, University of Zurich, Hirschengraben 84, 8001 Zurich, Switzerland

**Keywords:** Algorithm, Geriatrics, In-hospital mortality, Multiple trauma, Standard of care

## Abstract

**Background:**

The demographic changes towards ageing of the populations in developed countries impose a challenge to trauma centres, as geriatric trauma patients require specific diagnostic and therapeutic procedures. This study investigated whether the integration of new standard operating procedures (SOPs) for the resuscitation room (ER) has an impact on the clinical course in geriatric patients. The new SOPs were designed for severely injured adult trauma patients, based on the Advanced Trauma Life Support (ATLS) and imply early whole-body computed tomography (CT), damage control surgery, and the use of goal-directed coagulation management.

**Methods:**

Single-centre cohort study. We included all patients ≥65 years of age with an Injury Severity Score (ISS) ≥ 9 who were admitted to our hospital primarily via ER. A historic cohort was compared to a cohort after the implementation of the new SOPs.

**Results:**

We enrolled 311 patients who met the inclusion criteria between 2000 and 2006 (group *Pre*SOP) and 2010–2012 (group SOP). There was a significant reduction in the mortality rate after the implementation of the new SOPs (*P* = .001). This benefit was seen only for severely injured patients (ISS ≥ 16), but not for moderately injured patients (ISS 9–15). There were no differences with regard to infection rates or rate of palliative care.

**Conclusions:**

We found an association between implementation of new ER SOPs, and a lower mortality rate in severely injured geriatric trauma patients, whereas moderately injured patients did not obtain the same benefit.

**Trial registration:**

Clinicaltrials.gov NCT03319381, retrospectively registered 24 October 2017.

## Background

Populations continue to age in developed countries [1]. Switzerland exhibits the same demographic trends as other developed countries in Western Europe [2], where 18.1% of the Swiss population was aged ≥65 years in 2016, but people aged > 65 years comprised only 11.5% of the population in 1970 [3]. People are living longer and maintain independent and active lifestyles; associated with a higher proportion of geriatric patients [4]. Age is known to be a significant risk factor for morbidity and mortality in trauma patients [5]. Several studies have demonstrated that geriatric trauma patients have worse outcomes if the severity of the injury is equivalent [6–10]. These poor outcomes are linked to a higher susceptibility to post-traumatic infections, decreased physiological reserves, and pre-existing diseases in elderly trauma patients [11, 12]. This concept of frailty has only recently been recognized in surgical practice [13]. Anticoagulants make them more vulnerable to intracranial haemorrhage [14] and prolonged ventilatory support due to frailty is a risk factor for organ failure [15]. However, under-triage, a well-recognised phenomenon in geriatric trauma patients, might contribute to the poor outcome [16]. The American Geriatrics Society and John A. Hartford Foundation developed a research agenda to enhance the quality of care of geriatric patients including trauma-related questions [17]. We aimed to address the need for standardisation of definitions and evaluating the prognostic value of injury severity scores in improving outcomes in geriatric trauma patients.

The present study determined the outcomes for geriatric trauma patients aged over 65 years. In particular, we investigated whether there were changes in the in-hospital mortality, infection rate, and rate of palliative care (withdrawal of medical support) after the implementation of new standard operating procedures (SOPs) comprising early whole-body CT, damage control surgery, and the use of goal-directed coagulation management based on an Advanced Trauma Life Support (ATLS)-based algorithm. We included patients with an Injury Severity Score (ISS) ≥ 9 in order to determine whether moderately and severely injured geriatric trauma patients benefited from the implementation of the new SOPs.

## Methods

### Study design and patients

This study involved the analysis of a prospective single-centre database which bases upon the national trauma registry data of our single centre. The regional institutional review board approved this study (Kantonale Ethikkommision Zürich, Switzerland, StV-01/2008, 20.11.2007). The need for consent from patients was waived because the database was an anonymous registry. The present study was conducted in accordance with the principles of the Declaration of Helsinki and Good Clinical Practice Guidelines. Furthermore, this study adhered to the “STrengthening the Reporting of OBservational studies in Epidemiology” (STROBE) recommendations for cohort studies [18].

### Definitions

The primary endpoint of the study was in-hospital mortality, defined as trauma related death during the hospital course. Infections and rate of palliative care were secondary outcome parameters. Criteria for infection varied depending on the site of infection. Pneumonia was diagnosed when a predominant organism was isolated from appropriately obtained sputum cultures in the setting of purulent sputum production and/or a new or changing pulmonary infiltrate on chest radiography. Bloodstream infections were diagnosed when the predominant organism was identified in blood cultures obtained under sterile conditions. Criteria for urinary tract infections (UTIs) included isolation of > 10^5^ organisms/ml urine or > 10^4^ organisms in patients with symptoms typical for UTIs. Criteria for catheter-related infection included isolation of > 5 colony forming units (CFU) from catheter tips cultured only in the setting of suspected infection. Postoperative surgical side infection was said to be present in case of incision drainage or the presence of an abscess with at least one positive culture, as well as any delay in wound healing that was treated by antibiotics or surgical revision.

Palliative care was defined of withdrawal of medical support. Withdrawal of medical support was initiated in nonsurvivable injuries or unconsciousness patients with severe traumatic brain injury (TBI) after neurosurgical consultation and obviously serious brain damage in whom a severely disabled outcome is anticipated. However, withdrawal of medical support was only initiated following consultation of close family members.

### Parameters of interest

The recorded data comprised the age, gender, patient trauma load, and trauma sequelae according to the maximum injury severity on the Maximum Abbreviated Injury Scale 2005 Update 2008 (MAIS) for different body regions [19], ISS [20] and new ISS (NISS) [21], Glasgow Coma Scale (GCS) [22], base excess [23], lactate [24], haemoglobin [25], prothrombin (PT) [26], Acute Physiology and Chronic Health Evaluation (APACHE) II Score [27], and the Trauma and Injury Severity Score (TRISS) [28], where the latter was used to predict mortality. The standardized mortality ratio (SMR) was calculated as the observed mortality divided by the expected mortality.

### Group distribution

In our hospital, we use a standardised clinical approach to trauma patients according to leading trauma guidelines based on ATLS [29]. The implemented SOPs have additionally included early whole-body CT scans and consequent application of damage control surgery principles since 2008 [30]. In the same year, the first version of a goal-directed transfusion protocol was introduced. In 2009, other changes in trauma management have also been made, including early administration of tranexamic acid, restrictive fluid resuscitation, and permissive hypotension. Therefore, in the years before (2000–2006), and the years after (2010–2012) the introduction and full implementation of these changes were chosen for analysis and verified by internal controls. Details, especially our goal-directed transfusion protocol algorithm, were described previously [30]. Patients admitted via ER with need for intensive care treatment after the ER phase were included and compared before the implementation of the new SOPs (group *Pre*SOP; 1 January, 2000–31 December, 2006) and after their implementation in 2009 (group SOP, 1 January, 2010–31 December, 2012). The exclusion criteria comprised age < 65 years, ISS < 9, and transfers from other hospitals.

### Statistical analysis

Patients were classified into two groups according to the time period (group *Pre*SOP; time period 2000–2006; group SOP; time period 2010–2012). Descriptive statistics were calculated to summarize the characteristics of the study population. The data were represented as the mean ± standard deviation (SD) for continuous variables and as proportions for categorical variables. Person’s chi-square, Fisher’s exact and Mann–Whitney *U* test were used to compare the treatments.

Binary logistic regression analysis was conducted to measure the strengths of associations and to identify possible risk factors related to mortality: time period, ISS group, PT group, age, and gender. The Hosmer–Lemeshow goodness-of-fit test were used to test the quality of the logistic models. All analyses were two-sided and a *P*-value < .05 was considered to indicate a significant difference. All statistical analyses were performed with SPSS software (version 23.0; IBM Corporation, Armonk, NY, USA). Graphical visualizations were prepared using Excel and Visio Professional 2016 (Microsoft, Redmond, WA, USA).

## Results

**Fig. 1:**
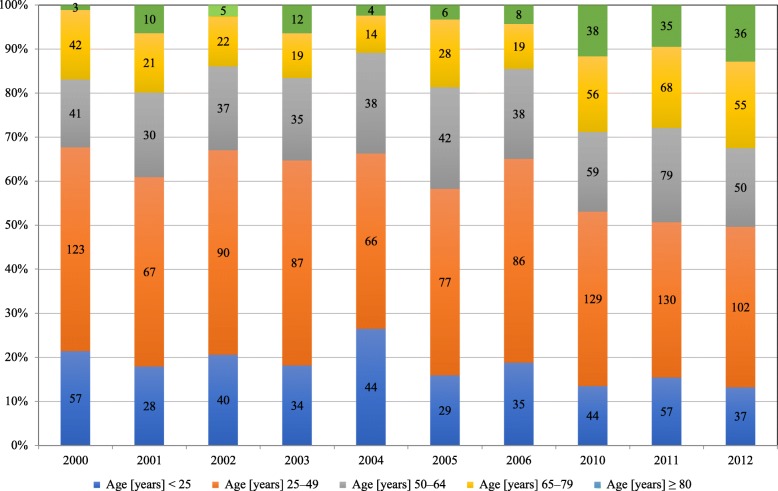
Percental proportions in age groups for all trauma patients. Absolute numbers within the bars

In recent decades, more trauma patients have been hospitalized in our clinic and the proportion of patients aged ≥65 years has increased (Fig. 1).

The characteristics and parameters for the patients are shown in Table 1. We analysed 311 patients who met the inclusion criteria in this study, i.e., 131 between 2000 and 2006 in group *Pre*SOP, and 180 between 2010 and 2012 in group SOP. There were significant differences between the cohorts in terms of age, lactate, APACHE II score, MAIS head or neck, MAIS abdomen, and MAIS external.

**Table 1:** Characteristics of patients in the two cohorts. Data represent the mean ± standard deviation and numbers (proportions). APACHE: Acute Physiology and Chronic Health Evaluation; AP: arterial pressure; GCS: Glasgow Coma Scale; ISS: Injury Severity Score; MAIS: Maximum Abbreviated Injury Scale in this region; NISS: New ISS; TRISS: Trauma and Injury Severity Score

Characteristics	*Pre*SOP (2000–2006)	SOP (2010–2012)	Total	*P*-value
N	131	180	311	
Age [years]	75 ± 7	77 ± 8	76 ± 7	.046*
Gender [% male]	59%	59%	59%	.91^**‡**^
MAIS head or neck	3.02 ± 2.07	3.72 ± 1.91	3.43 ± 2.00	.032*
MAIS face	0.45 ± 0.94	0.66 ± 1.07	0.57 ± 1.02	.16*
MAIS spine	0.64 ± 1.28	0.64 ± 1.20	0.64 ± 1.23	.33*
MAIS chest	1.75 ± 1.76	1.51 ± 1.73	1.61 ± 1.74	.28*
MAIS abdomen	0.83 ± 1.56	0.37 ± 0.98	0.56 ± 1.27	.007*
MAIS pelvis	0.71 ± 1.29	0.63 ± 1.34	0.66 ± 1.32	.48*
MAIS extremities	1.36 ± 1.49	1.18 ± 1.45	1.25 ± 1.47	.69*
MAIS external	0.36 ± 0.63	0.74 ± 0.88	0.59 ± 0.81	.008*
GCS	7.31 ± 5.32	8.18 ± 5.15	7.81 ± 5.23	.39*
ISS	29 ± 12	37 ± 24	34 ± 20	.28*
NISS	42 ± 17	42 ± 22	42 ± 20	.41*
Mean AP [mmHg]	93 ± 25	97 ± 24	96 ± 24	.25*
Base excess [mEq/L]	−4.3 ± 5.0	− 3.5 ± 5.6	−3.8 ± 5.4	.64*
Lactate [mmol/L]	3.5 ± 2.8	2.3 ± 2.0	2.8 ± 2.5	<.001*
Haemoglobin [g/L]	10.4 ± 4.2	11.6 ± 7.8	11.2 ± 6.7	.094*
Prothrombin [% normal]	76 ± 24	71 ± 25	72 ± 25	.072*
APACHE II score	21 ± 9	18 ± 8	20 ± 8	.034*
TRISS	0.71 ± 0.28	0.63 ± 0.39	0.66 ± 0.35	.72*

* Mann–Whitney U test, ^**‡**^Pearsons’s chi-square

**Fig. 2:**
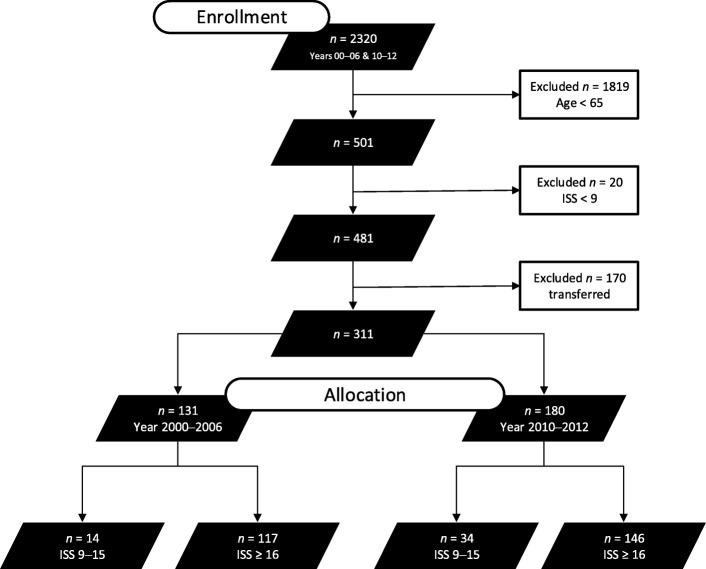
Flow chart illustrating the patient inclusion and exclusion criteria

Using the ISS, patients were grouped into moderately (ISS = 9–15) and severely (ISS ≥ 16) injured subgroups. During 2000–2006 (group *Pre*SOP), 14 patients were enrolled into the ISS = 9–15 subgroup and 117 in the ISS ≥ 16 subgroup. Between 2010 and 2012 (group SOP), 34 patients were moderately injured and 146 were severely injured (Fig. 2).

**Fig. 3:**
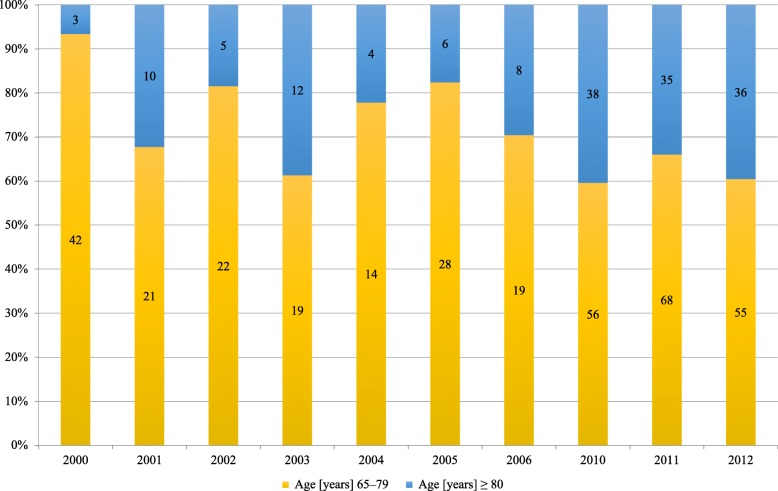
Percental proportions in age groups for study population. Absolute numbers within the bars

Patients aged ≥65 years were further divided into 65–79 years and ≥ 80 years subgroups. Figure 3 shows the increase in the proportion of patients aged ≥80 years during the study period.

In group *Pre*SOP, 84/131 of the geriatric trauma patients died (64.1%), 28 patients suffered from infections (21.4%), and 31 patients (23.7%) received palliative care. However, information about the withdrawal of medical support was missing for six patients. Among the 84 patients who died, four were moderately injured (ISS = 9–15) and 80 were severely injured (ISS ≥ 16). Therefore, the mortality rate was 28.6% (*n* = 4/14) in the ISS = 9–15 subgroup. The mortality rate was more than twice as high in the ISS ≥ 16 subgroup with a mortality rate of 68.4% (*n* = 80/117). The infection rate in the ISS = 9–15 subgroup was 7.1% (*n* = 1/14) in group *Pre*SOP. However, the infection rate in the ISS ≥ 16 subgroup was 23.1% (*n* = 27/117) in the same time period.

In group SOP, the mortality rate was significant lower (44.4%; *n* = 80/180; *P* = .001) whereas the rate of infections (21.9%; *n* = 39) and withdrawal of medical support (28.3%; *n* = 51) was comparable and not significant different with group *Pre*SOP. Among the 80 patients who died, again only seven belonged to the ISS = 9–15 subgroup and the majority of 73 to the ISS ≥ 16 subgroup as in group *Pre*SOP. The mortality rate was with 20.6% (*n* = 7/34) lower but not significant in the ISS 9–15 subgroup (*P* = .71). The mortality rate was again two and a half times higher at 50% (*n* = 73/146) in the ISS ≥ 16 subgroup and significant lower to group *Pre*SOP (*P* = .003). The infection rate was higher but not significant with 14.7% (*n* = 5/34) in the ISS = 9–15 subgroup in group SOP compared to group *Pre*SOP and not significant similar in the ISS ≥ 16 subgroup (23.6%; *n* = 34/144). Information about the infection status was missing for two patients.

The SMR was 0.90 in group *Pre*SOP and 0.70 in Group SOP. Hence, the observed mortality rate was below the expected mortality rate in both study periods.

The mean PT in the 259 patients was 72.5% (SD = 24.9%, range = 10–136%). The mortality rate was 80% (*n* = 8/10) in group *Pre*SOP among patients with PT ≤ 30%, which probably indicated pre-existing anticoagulation medication. In group SOP, the mortality rate for patients with PT ≤ 30% was 53.3% (*n* = 8/15), which was not significant (*P* = .23). There was a significant (*P* = .002) lower mortality rate of patients with PT > 30% from 60.5% (*n* = 49/81) in group *Pre*SOP to 39.2% (*n* = 60/153) in group SOP. In group *Pre*SOP, 10% of the patients (*n* = 1/10) with PT ≤ 30 and 18.5% of the patients (*n* = 15/81) with PT > 30% suffered from infections. In group SOP, 46.7% of the patients (*n* = 7/15) with PT ≤ 30 and 21.2% of the patients (*n* = 32/151) with PT > 30% suffered from infections. Therefore, between the two-time periods, there was a not significant trend of increase in the infection rate among patients with PT ≤ 30% (from 10.0 to 46.7%; *P* = .088) but a not significant increase among patients with PT > 30% (from 18.5 to 21.2%; *P* = .63).

TBIs were the leading cause of death in both time periods (60.2% of deaths in time group *Pre*SOP and 72.5% of deaths in group SOP, respectively, which corresponded to a higher MAIS head or neck score in group SOP). However, the rate of exsanguinating patients decreased from 26.5% in group *Pre*SOP to 7.5% in group SOP.

Table 2 shows the statistics for the mortality rate and infection rate.

**Table 2:** Differences in mortality and infection rates between 2000 and 2006 (group *Pre*SOP) and 2010–2012 (group SOP)

Outcome	Subgroup	*Pre*SOP [n] / %	SOP [n] / %	*P*-value
Mortality rate	ISS = 9–15	4 / 28.6%	7 / 20.6%	.71*
	ISS ≥ 16	80 / 68.4%	73 / 50.0%	.003^‡^
	Total	84/ 64.1%	80/ 44.4%	.001^‡^
	PT ≤ 30%	8 / 80.0%	8 / 53.3%	.23*
	PT > 30%	49 / 60.5%	60 / 39.2%	.002^‡^
Infection rate	ISS = 9–15	1 / 7.1%	5 / 14.7%	.66*
	ISS ≥ 16	27 / 23.1%	34 / 23.6%	.92^‡^
	Total	28 / 21.4%	39 / 21.9%	.91^‡^
	PT ≤ 30%	1 / 10.0%	7 / 46.7%	.088*
	PT > 30%	15 / 18.5%	32 / 21.2%	.63^‡^

*ISS* Injury Severity Score, *PT* prothrombin

*Fisher’s exact, ‡Pearsons’s chi-square

Multivariate binary logistic regression indicated that time period, ISS group, and age were all independently associated with in-hospital death. PT group and gender were not associated with in-hospital death. The Hosmer–Lemeshow test (chi-square = 13.156, *P* = .11) indicated that the numbers of deaths were not significantly different from those predicted by the model, and thus the overall model fit was fair.

**Fig. 4:**
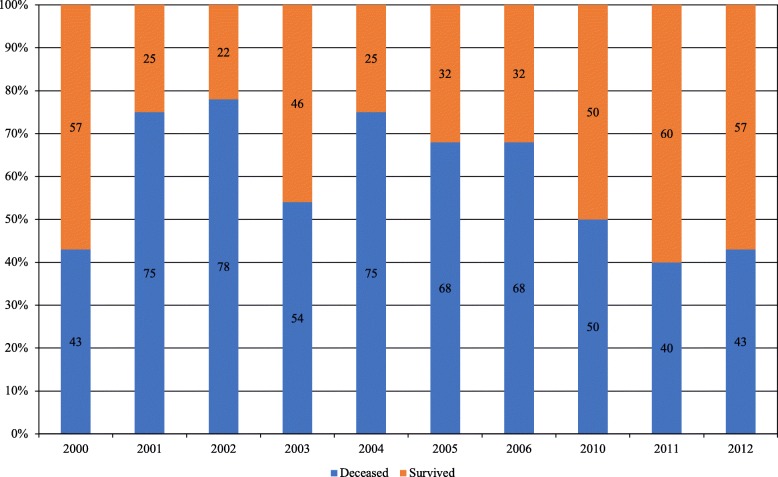
Percental proportions of mortality for study population. Percentage within the bars

Multivariate binary logistic regression indicated that age was independently associated with infection, whereas time period, ISS group, PT group, and gender were not associated with infection. The Hosmer–Lemeshow test (chi-square = 3.685, *P* = .88) indicated that the numbers of infections were not significantly different from those predicted by the model, and thus the overall model fit was good. There was no obvious linear trend over time, neither for mortality nor for infection (Tables 3, 4 and Fig. 4).

**Table 3:** Logistic regression model of mortality and infection rates. Time period 2000–2006 and 2010–2012, ISS: Injury Severity Score (grouped ISS 9–15 and ISS ≥ 16); PT: prothrombin (grouped PT > 30% and ≤ 30%)

Outcome	Parameter	Regression coefficient β	*P*-value	Odds ratio (e^β^; 95% CI)
Mortality rate	Time period	−1.05	<.001	.35 (.20–.62)
	ISS group	1.51	<.001	4.54 (2.05–10.08)
	PT group	−.68	.15	.51 (.20–1.28)
	Age [years]	.073	<.001	1.08 (1.04–1.12)
	Gender	.442	.12	1.56 (0.89–2.72)
Infection rate	Time period	0.51	.13	1.67 (.86–3.26)
	ISS group	0.81	.088	2.24 (.89–5.68)
	PT group	−.77	.11	0.46 (.18–1.17)
	Age [years]	−.05	.030	.95 (.91–.96)
	Gender	1.49	.86	.94 (.50–1.79)

**Table 4:** The predictivity of time period for survival and infection by binary logistic regression with the possible confounders. With and without estimated linear trend over time. ISS: Injury Severity Score; CI: confidence interval

Parameter	Wald	*P*-value	Odds ratio	95% CI
a. Survival with estimated linear trend over time				
Period	6.730	.009	.325	.139–.760
ISS group	15.990	.000	4.604	2.178–9.732
Age [years]	17.621	.000	1.076	1.040–1.113
Trend	.290	.590	1.045	.889–1.229
Constant	15.292	.000	.002	
b. Survival without estimated linear trend over time				
Period	13.331	.000	.392	.237–.648
ISS group	16.038	.000	4.613	2.183–9.748
Age [years]	17.645	.000	1.075	1.040–1.113
Constant	19.440	.000	.001	
c. Infection with estimated linear trend over time				
Period	.516	.472	.716	.288–1.781
ISS group	2.987	.084	2.242	.897–5.600
Age [years]	5.833	.016	.953	.917–.991
Trend	1.891	.169	1.140	.946–1.373
Constant	.603	.437	4.201	
d. Infection without estimated linear trend over time				
Period	.420	.517	1.205	.686–2.115
ISS group	2.997	.083	2.240	.899–5.582
Age [years]	5.727	.017	.954	.917–.991
Constant	.085	.770	1.661

## Discussion

Integration of guidelines and SOPs have been shown to improve in the hospital course, and clinical outcome [31], including a lower rate of mortality and better clinical outcomes in severely injured patients [32, 33]. The integration of whole-body CT scans into the early resuscitation phase for patients with major trauma has increased the probability of survival in several studies [34–36]. In contrast, the international, multicentre, randomised controlled REACT-2 trial found no advantage of an immediate whole-body CT scans regarding in-hospital mortality, but there are some discussions about the weaknesses of this study, like a high number of not severely injured patient, a high dropout rate, or a high number of cross-overs from the standard work-up group to the CT-group [37–39]. Especially the fact that many patients with a standard ATLS-based work-up will have a CT scan later could also be shown by our research group [40]. Furthermore, the use of a restricted volume replacement strategy during initial resuscitation has been proposed [41, 42]. In addition, the damage control approach has become the standard for the care of patients with multiple injuries [43–45].

In the present study, we explored the effects on the mortality and infection rates in geriatric trauma patients following the implementation of new ER SOPs. The main findings of this study were that the mortality rates decreased but the infection rates were unchanged after the implementation of the new SOPs. In addition, the mean age and mean ISS increased in the study population in recent years.

The synchronous implementation of different SOPs made it difficult to determine the individual impact of each. Other therapeutic changes might have occurred during hospital stays, which could have influenced the outcome parameters and we did not assess the long-term outcomes for the elderly trauma patients. We acknowledge that long-term outcome is an important measure in geriatric trauma care because in-hospital mortality underestimates post-discharge mortality; and trauma in the elderly affects long-term survival and health-care utilization [46–48]. However, there is growing evidence that interdisciplinary care of the hospitalised elderly trauma patient improves outcomes and reduces costs [49, 50]. A single centre study from the United Kingdom showed a significant improvement in mortality and quality of care indicators after implementation of orthogeriatric care in patients with a hip fracture [51]. Good clinical outcomes in geriatric trauma patients are based upon several disciplines and the management in the resuscitation room is only one aspect of the recovery process. Continuity of care is especially important for these patients [52].

Patients with an ISS = 9–15 might have been under-represented because they were probably not triaged to the ER, and thus were not included in the database. It is important to note that there were differences in the characteristics of the patients in our two study cohorts. In the Group SOP, the patients were slightly older, with higher MAIS head or neck and external, lower lactate on admission, lower APACHE II, and lower MAIS abdomen scores. The continuous increase in the age of trauma patients agrees with data obtained from the German Trauma Registry, which showed that there was an increase in patient age over recent decades [53], although the not significant tendency of increase in the ISS in our study did not agree with previous data. This difference is probably explained by the fact that we act as a referral centre for severe trauma, and especially for severe TBI, which was demonstrated by the increase in the MAIS head or neck score. In recent years in our country the medical care for these patients was more and more centralized.

The implementation of new SOPs into clinical practice is a complex and time-consuming process. This process was observed closely using written instructions and regular training sessions within our resuscitation team using simulation-based training and video review. Therefore, we think that our results are valid, because we chose an intermediate period of 4 years between both groups for implementation, which has been proofed in internal controls and some other studies of our research group [30]. Therefore, we can assure a strict implementation of the new SOPs in the Group SOP.

Our decrease in the mortality rate in geriatric trauma patients is consistent with the findings of Schoeneberg et al. who reported a similar reduction in mortality in severely injured patients (ISS ≥ 16) after the introduction of evidence-based guidelines [33]. The higher probability of survival could be explained partly by a decrease in TRISS. However, the SMR decreased from group *Pre*SOP to group SOP, although the SMR was below 1 in both time periods (0.90 and 0.70, respectively), thereby indicating a better outcome than expected. In addition to severely injured trauma patients (ISS ≥ 16) who have been investigated in many previous studies, we included moderately injured patients (ISS = 9–15) in order to assess this under-represented patient group. For a geriatric trauma patient, an ISS ≥ 9 may represent severe trauma, as for example a hip fracture has an ISS of 9. However, literature on low level falls and this population of moderately injured geriatric trauma patients is limited [6, 54]. In our study, patients with an ISS ≥ 16 showed a decrease in mortality rate after implementation of the new SOPs, but the decrease in the mortality rate was not significant for patients with an ISS = 9–15. The reasons for this difference are unclear, but we consider that in this moderately injured patient group, survival may have depended on factors other than the implementation of the new SOPs comprising early whole-body CT, damage control surgery, and goal-directed coagulation management. The new SOPs aimed to facilitate the prompt detection of all injuries, especially haemorrhage. Minor TBIs, non-displaced rib, or pelvic fractures can often be detected in elderly trauma patients, and more research is needed to improve the survival rates of these patients.

The mortality rates determined for geriatric trauma patients in the present study are higher than those reported in other studies [10, 53, 55, 56]. It should be noted that there are significant differences in geriatric trauma outcomes between trauma centres [57–59]. However, our hospital acts as a referral centre for severe trauma and TBI, and thus the injury severity in our patients might have been higher than that in other study populations. The overall mean ISS of 34 (± 20) reflects the injury severity in our study population. Furthermore, we did not exclude patients for whom medical support was withdrawn. In both time periods, the rate of palliative care was around 25%. In addition, the age threshold that should be used to define elderly trauma patients is still controversial [60]. Thus, setting the age cut-off at different levels might have changed the mortality rates in the subgroups. We selected 65 years as the age threshold for geriatric trauma patients because of several reasons. First, large study cohorts have used the same threshold, which facilitates comparisons with other studies [7, 9, 55]. Furthermore, many epidemiological studies have employed an age cut-off of 65 years in Switzerland because it is the current retirement age. Thus, we consider that 65 years is a practical cut-off age for geriatric trauma patients.

The patients in our study cohort with PT > 30% exhibited a marked increase in their probability of survival after the implementation of the new SOPs. Stein et al. also found that the 24-h and in-hospital mortality decreased significantly after the implementation of goal-directed coagulation management [30], although they noted that their study lacked sufficient power to assess this endpoint. It is not clear why the mortality rate of patients with PT ≤ 30% did not improve in the same manner in our study, but we assume that patients with PT ≤ 30% (which probably indicates anticoagulation medication) were already being treated aggressively before the implementation of goal-directed coagulation management because of known pre-existing anticoagulation medication. However, patients with PT ≤ 30% did not appear to have significantly worse outcomes.

In our study, the infection rate did not change after the implementation of the new SOPs despite a tendency of a higher infection rate in patients with a PT ≤ 30%. It is well known that the immune system function of geriatric trauma patients is impaired and they are highly susceptible to infections [11]. Bochicchio et al. reported that age increased the risk of nosocomial infections in trauma patients aged ≥65 years, who had a significantly higher mortality compared with younger patients, whereas respiratory infections were the most common followed by genitourinary infections in their study [61]. These results are consistent with our findings where pneumonia was the most common infection. However, in a multicentre cohort study, Blot et al. showed that ventilator-associated pneumonia did not occur more frequently among the elderly, although the associated mortality was higher in these patients [62]. In a review, Hazeldine et al. demonstrated that age-related changes in immune function may contribute to poor outcomes for geriatric trauma patients [63]. Therefore, further research is required to prevent infections and improve the outcomes for infected geriatric trauma patients.

This study had several limitations and strengths. It was based on a retrospective review of a prospectively collected single-centre trauma database. Registry data must be taken with caution as they only can show association and not cause-effect relationships. However, our data was collected and analysed by well-instructed personnel with an internal and external quality control. This was done one the one hand by the senior author in case of coding questions and reviewing all cases personally and on the other hand by the national trauma registry by its structure with quality control algorithms. Because of the single-centre design, the results are only applicable to our trauma centre. It was a database investigation with a prospective data collection not specifically selected for this study, which allows the possibility of bias from unmeasured confounders associated with comorbidities and frailty. Undetected confounding factors, such as changes in prehospital treatment, may exist and must be considered when interpreting our results. Using smaller time increments could reduce the impact of undetected confounding factors. However, smaller time increments would reduce the number of patients and therefore the statistical power and increase failure to detect secular trends. Furthermore, we used in-hospital mortality as our end marker without any follow up data.

## Conclusions

Our main findings suggest that the implementation of new SOPs comprising early whole-body CT, damage control surgery, and the use of goal-directed coagulation management significantly reduced the mortality rate in severely injured geriatric trauma patients, whereas moderately injured patients seemed not obtain the same benefit and with no influence on the infection rate. Further research is needed to improve the outcomes for this fast-growing population.

## Data Availability

All data of this submission are available from the Dryad Digital Repository, please consider the following link: 10.5061/dryad.2v6wwpzhk

## Abbreviations

AIS: Abbreviated Injury Scale
APACHE: Acute Physiology and Chronic Health Evaluation
ATLS: Advanced Trauma Life Support
CT: Computed tomography
ER: Resuscitation room
GCS: Glasgow Coma Scale
ISS: Injury Severity Score
MAIS: Maximum Abbreviated Injury Scale
NISS: New Injury Severity Score
PT: Prothrombin
SD: Standard deviation
SMR: Standardized mortality ratio
SOP: Standard operating procedure
TBI: Traumatic brain injury
TRISS: Trauma and Injury Severity Score
